# Firm smooth polypoid nodule within a skin graft

**DOI:** 10.1016/j.jdcr.2023.08.024

**Published:** 2023-08-30

**Authors:** Sarah Williamson, Stephen Somach

**Affiliations:** Department of Dermatology, MetroHealth Medical Center, Cleveland, Ohio

**Keywords:** elephantiasis, localized lymphedema, nodule, pseudotumor, skin graft, thigh

## Presentation

**Fig 1:**
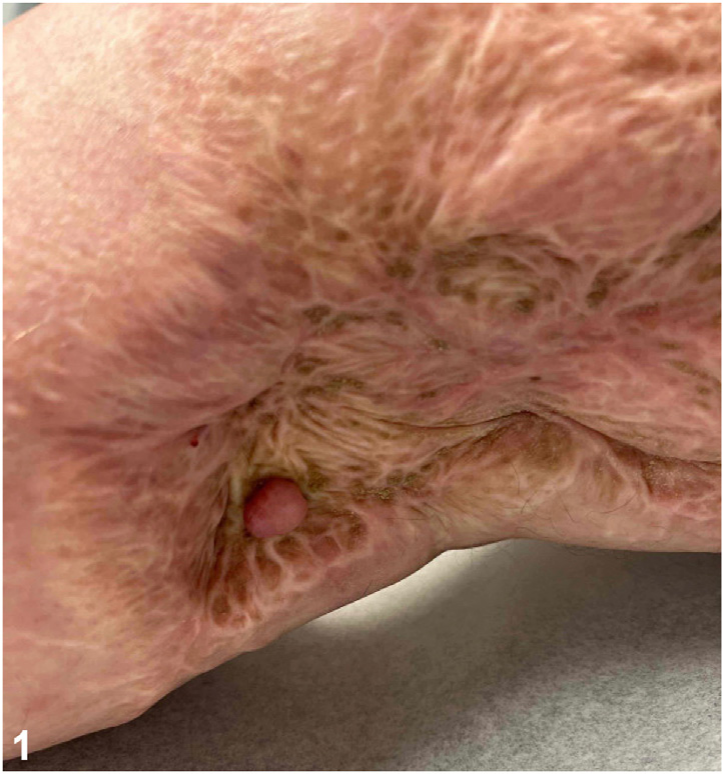

**Fig 2:**
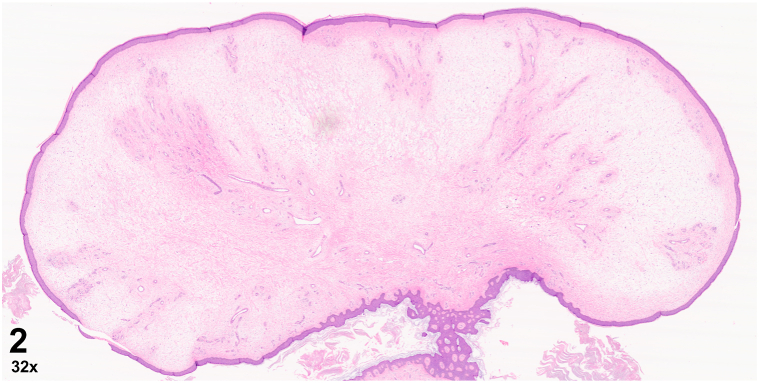

**Fig 3:**
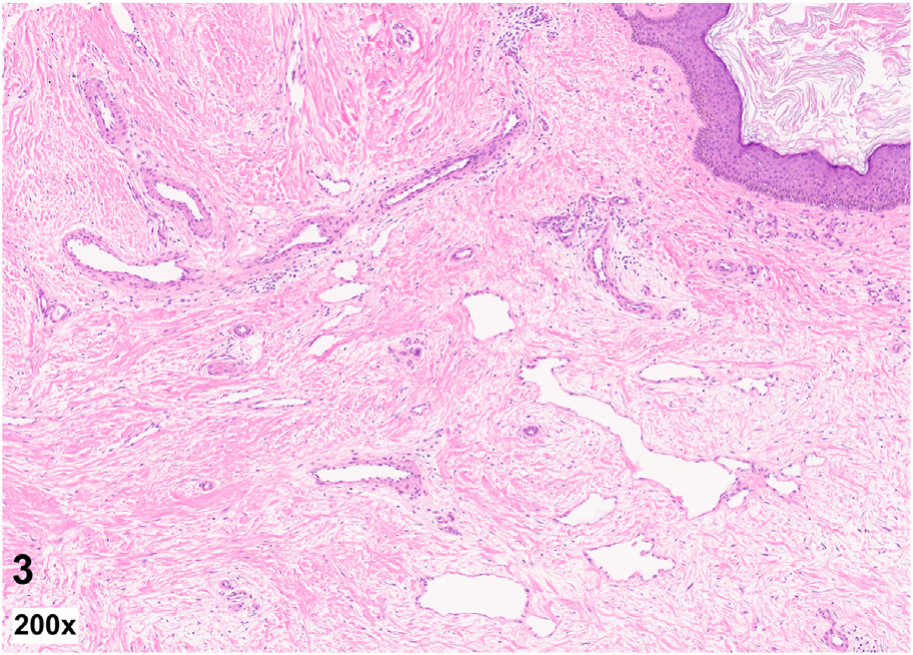

A 58-year-old obese man with a history of renal cell carcinoma presented with an asymptomatic pink firm smooth polypoid nodule on the right medial thigh which had slowly enlarged over several months. He had extensive trauma to the right lower extremity 30 years prior following a train accident. The nodule was located within an indurated fibrotic plaque at the site of a prior skin graft (Fig 1). Histopathology revealed dermal angioplasia with edema, lymphectasia, fibroplasia, scant chronic inflammation, and mildly increased mucin (Figs 2 and 3).

**Question 1: What is the most likely diagnosis?**

A. Nevus lipomatosis superficialis
B. Nodular lymphedema
C. Keloid
D. Nodular hidradenoma
E. Cutaneous metastasis of renal cell carcinoma

**Answers:**

A. Nevus lipomatosis superficialis – Incorrect. This is a benign hamartoma that presents as soft grouped papules and nodules. The characteristic histopathologic finding is mature adipocytes located within the dermis.
B. Nodular lymphedema – Correct. This patient had a history of chronic lymphedema of the right lower extremity secondary to his prior trauma, and his enlarging polypoid nodule was most consistent with nodular lymphedema. The diagnosis was made based on the histopathologic evaluation which revealed features of lymphedema, and he required no further treatment over the next few months after removal of the nodule.
C. Keloid – Incorrect. Keloids can similarly present as firm smooth nodules within areas of prior trauma but are composed of thick hyalinized eosinophilic collagen fibers.
D. Nodular hidradenoma – Incorrect. Nodular hidradenomas are also well-circumscribed nodules which can affect the limbs. However, they are adnexal tumors of eccrine or apocrine origin with solid and cystic components evident upon histopathologic examination.
E. Cutaneous metastasis of renal cell carcinoma – Incorrect. Despite the patient’s prior history of renal cell carcinoma, the histopathologic examination did not show the typical features of clear cell adenocarcinoma with tumor cells filled with abundant clear cytoplasm.

**Question 2: A biopsy specimen from a patient with this condition would be highlighted with which special stain?**

A. AE1/AE3
B. PAX8
C. CK20
D. S100
E. D2-40

**Answers:**

A. AE1/AE3 – Incorrect. AE1/AE3 are a mixture of antibodies to low- and high-molecular weight cytokeratins. This pan-cytokeratin stain would identify an epithelial tumor and would not highlight a cutaneous nodule composed of lymphedema.
B. PAX8 – Incorrect. PAX8 staining would be positive in metastatic renal cell carcinoma, not in nodular lymphedema.
C. CK20 – Incorrect. CK20 positivity can be seen in a paranuclear dot pattern in Merkel cell carcinoma and in metastases from certain adenocarcinomas. CK20 would not highlight nodular lymphedema.
D. S100 – Incorrect. S100 stains neural crest-derived cells. The adipocytes in a nevus lipomatosus superficialis would be highlighted by S100, but nodular lymphedema would not be S100 positive.
E. D2-40 – Correct. D2-40 is an antibody that binds to podoplanin, a mucin-type glycoprotein expressed by lymphatic endothelial cells. In our case, D2-40 staining revealed prominent ectatic lymphatic vessels.

**Question 3: Which statement is correct regarding localized lymphedema (LL)?**

A. It can occur as a complication of breast cancer treatment
B. Affected patients tend to have normal body mass index
C. It usually presents as a discrete polypoid nodule
D. It is an inherited condition in most patients
E. It rarely affects the lower extremity

**Answers:**

A. It can occur as a complication of breast cancer treatment – Correct. LL of the breast has been described as a complication after surgery to treat breast cancer.^1^
B. Affected patients tend to have normal body mass index – Incorrect. LL has been described often as a complication of obesity and in this setting usually occurs as massive LL.^2^^,^^3^ It has been hypothesized that excess adipose tissue can compress lymphatic vessels, leading to localized lymphedema masses.^2^
C. It usually presents as a discrete polypoid nodule – Incorrect. Lu, et al reported 11 cases of solitary lymphedema polyps, each involving the anogenital region,^4^ and our case adds to these as an additional report of a singular polypoid nodule. However, numerous prior cases of LL have described the clinical presentation as confluent grouped polyps or large papillomatous plaques.^2^, ^3^, ^4^
D. It is an inherited condition in most patients – Incorrect. LL is typically an acquired secondary form of edema due to impaired lymphatic drainage in the setting of trauma, surgery, radiation, infection, and obesity.^5^
E. It rarely affects the lower extremity – Incorrect. In the original case series of massive LL by Farshid and Weiss, 12 of the 14 described patients had massive LL of the thigh.^2^

## Conflict of interest

None disclosed.
